# Motor function score changes in severe 5q spinal muscular atrophy during risdiplam treatment: an observational longitudinal nationwide cohort study

**DOI:** 10.1016/j.eclinm.2026.104008

**Published:** 2026-06-30

**Authors:** Lina M. Vermeer, Fay-Lynn Asselman, Ruben P.A. van Eijk, Inge Cuppen, Danny R. van der Woude, Bea M.H.E.A. Visser–de Heus, Saskia M.J. Hogervorst, Thijs J. Ruyten, Bart Bartels, Renske I. Wadman, W. Ludo van der Pol

**Affiliations:** a Department of Neurology & Neurosurgery, University Medical Centre Utrecht, UMC Utrecht Brain Centre, Utrecht University, Utrecht, the Netherlands; b Biostatistics and Research Support, Julius Centre for Health Sciences and Primary Care, University Medical Centre Utrecht, Utrecht University, Utrecht, the Netherlands; c Child Development and Exercise Centre, Wilhelmina Children's Hospital, University Medical Centre Utrecht, Utrecht University, Utrecht, the Netherlands; d Department of Rehabilitation, Physical Therapy Science & Sports, UMC Utrecht Brain Centre, University Medical Centre Utrecht, the Netherlands

**Keywords:** Spinal muscular atrophy, Risdiplam, Disease modifying therapy, Motor function score, RULM, ATEND

## Abstract

**Background:**

Spinal muscular atrophy (SMA) is caused by the loss of function of the *SMN1* gene resulting in deficiency of intracellular survival motor neuron (SMN) protein and is characterised by progressive motor function loss. Motor function changes during treatment with *SMN2* splicing modifiers (i.e., nusinersen and risdiplam) have been shown in randomised clinical trials in infants, children and young adults with SMA, but not in severely affected adult patients, for whom risdiplam is often the only treatment option.

**Methods:**

Patients were screened for eligibility for risdiplam treatment between January and July 2021. We longitudinally evaluated motor scores during treatment up to 36 months using the Revised Upper Limb Module (RULM), the Adapted Test of Neuromuscular Disorders (ATEND), and hand strength tests. We assessed patient-reported Global Impression of Change (PGIC) after long-term treatment. Additionally, we investigated construct validity and responsiveness of ATEND as an additional outcome measure for severely affected patients.

**Findings:**

In this nationwide observational study, we analysed 72 patients with SMA types 1c and 2 (median age 29, IQR 23–42 years). We observed stabilised or improved RULM scores in 26 (43%) and increased ATEND scores in 33 (60%) patients after a median of 36 months of treatment. Eleven patients (18%) had a RULM score of 0 throughout treatment. ATEND score correlated strongly with RULM score supporting construct validity. After more than 3 years of treatment, 50 (89%) patients self-reported stability or improvement in overall wellbeing on the PGIC scale.

**Interpretation:**

Patients with SMA types 1 and 2 and severe motor impairment treated with risdiplam showed a deviation of the natural disease course of progressive motor score decline. The ATEND score is useful to monitor motor function change in severely affected patients. The majority of patients report improvement or stabilisation in motor function and overall wellbeing.

**Funding:**

None.

**Research in context**

**Evidence before this study**

Efficacy of risdiplam, a small molecule with *SMN2* splicing modifying properties, has been shown in a randomised placebo-controlled trial that allowed inclusion of symptomatic children, adolescents and young adults with spinal muscular atrophy (SMA) type 2 and 3. Post-marketing cohort studies have confirmed efficacy in symptomatic and presymptomatic infants. We searched Embase and PubMed for randomised controlled trials or cohort studies (n > 5) written in English, using the search terms ‘spinal muscular atrophy’ or ‘SMA’ and ‘risdiplam’ or ‘Evrysdi’ or ‘RG7916’ or ‘RO7034067’ and available corresponding MeSH or Emtree terms, up to April 1, 2026. We identified six studies with data on motor function changes during 6–31 months of treatment with risdiplam in adult patients who were not previously treated with other survival motor neuron (SMN) protein-targeting therapies. These (small) cohort studies reported motor function changes in adult patients with SMA type 1, 2, 3 and 4 after 9, 12, 24 or 31 months of risdiplam. One by Parmova and colleagues reported stabilisation of motor function for up to 36 months in a subset of patients with SMA type 2 (n = 11 at 36 months). However, (long-term) treatment results remain uncertain in (adult) patients with severely affected motor function at initiation of treatment.

**Added value of this study**

This longitudinal cohort study is the first to systematically assess long-term motor function changes during risdiplam use in severely affected patients. It provides real-world evidence on treatment for patients with SMA type 1 and 2 who are ineligible for other disease-modifying treatment options, such as onasemnogene abeparvovec (due to age or weight restrictions) or intrathecal nusinersen (due to severe scoliosis or previous scoliosis surgery). In addition, we report on the challenges of monitoring motor function in patients with long-standing SMA and severe motor impairment. Moreover, we show the added value of the Adapted Test of Neuromuscular Disorders (ATEND), a new motor function scale, and patient-reported outcome measures in capturing functional changes during treatment.

**Implications of all the available evidence**

We observed improving and stabilising motor function scores during risdiplam use in at least 43% patients with SMA type 1 and 2 and severe impairments. The Revised Upper Limb Module (RULM) and ATEND scores strongly correlated, but the ATEND scale added discriminatory Power at the lower end of the RULM scale, thus showing construct validity and responsiveness. In addition to objective motor function score improvement and stability, most patients reported improved overall wellbeing and motor function after more than 3 years of treatment.

## Introduction

Spinal muscular atrophy (SMA) is a hereditary neuromuscular disease caused by loss of function of the *SMN1* gene on chromosome 5q13. This results in deficiency of the survival motor neuron (SMN) protein and loss of alpha-motor neurons located in the spinal cord, but also in alterations in axons, the neuromuscular junction and muscle fibres.^1^, ^2^, ^3^, ^4^ SMA is characterised by variation in age at onset and progressive muscle weakness that predominates in proximal and axial muscle groups. This causes stunted gross motor development in the first months to years in the large majority of patients.^5^^,^^6^ Classification of SMA severity is based on the highest achieved motor milestone, i.e., whether or not (SMA type 1) children acquired the ability to sit (SMA type 2) and walk (SMA type 3) independently, and age at onset. Adult-onset SMA is classified as SMA type 4.

This broad range of severity is partially explained by copy number variation (1–5) of *SMN2* that crucially differs from *SMN1* in a C to T point mutation in exon 7 and therefore produces only small amounts of full-length SMN protein.^7^ *SMN2* mRNA is the target of the splicing modifying drugs nusinersen and risdiplam.^8^ Their efficacy has been shown in randomised control trials (RCTs) in symptomatic infants, children and young adults.^9^ Post-marketing studies have confirmed long-term efficacy and efficacy in presymptomatic infants and in subgroups not included in RCTs (Supplementary material I, Supplementary Table S1),^10^^,^^11^ with the important exception of severely affected adult patients (i.e., adolescents and adults with SMA types 1c and 2) with long disease duration with no access to other treatment regimens.

Assessing motor function changes in adult patients with SMA type 1c and 2 is complicated by the lack of adequate motor scales to assess motor function (Supplementary Table S1). Validated scales that have been used previously in RCTs include the Hammersmith Functional Motor Scale–Extended (HFMSE), Motor Function Measure, Revised Hammersmith Scale, 6-Minute Walk Test and the Revised Upper Limb Module (RULM), are not suited for patients with limited motor function because of their intrinsic floor effect.^12^

The aim of this observational study was therefore to monitor motor function changes during risdiplam use by adolescent and adult patients with SMA type 1c and 2 with severe weakness and to evaluate construct validity and responsiveness of the Adapted Test of Neuromuscular Disorders (ATEND).^13^ We analysed changes in motor ability by means of the RULM and evaluated the value of ATEND, (pinch) grip strength and patient-reported outcome measures (PROMs) as additional outcome measures

## Methods

### Study design and participants

We conducted a longitudinal observational population-based cohort study of patients with SMA type 1c and 2 in the Netherlands (population: 18 million) who were eligible for treatment with risdiplam through a compassionate use program (CUP).^14^ We assessed treatment efficacy over the course of approximately 3 years in all patients. We screened patients for eligibility between January and July 2021, except for four patients enrolled between July and August 2020 through a named-patient program. We collected medical and functional data as part of standard care at the outpatient clinic for neuromuscular diseases of the University Medical Centre of Utrecht (UMCU). We reported this study according to the Strengthening the Reporting of Observational Studies in Epidemiology (STROBE) criteria.

Patients were eligible for treatment with risdiplam if 1) their clinical diagnosis of SMA type 1 or 2 had been genetically confirmed (loss of function of *SMN1*) 2) they were not eligible for other genetic therapy options at time of screening (i.e., nusinersen and onasemnogene abeparvovec), and 3) they adhered to standards of care.^15^^,^^16^ Reasons for inability to treat with nusinersen were either inaccessibility for intrathecal treatment due to severe scoliosis or spondylodesis, or other contra-indications for (repeated) intrathecal injections. Relative contraindications to start treatment with risdiplam were cardiac disease, retinal abnormalities, use of medications with known interactions (i.e., MATE-1 substrate medication (metformin, dofetilide and procanamide), midazolam and itraconazole). Patients could not start treatment if they were pregnant or had a pregnancy wish for the next year.

All patients visited the Netherlands SMA centre at the outpatient clinic of neuromuscular diseases of the UMCU. Treatment with disease-modifying drugs (DMT) is centralised in the Netherlands. We reached out to patients using the national SMA database (which includes contact details of >90% of persons with SMA) and through the patient organisation. We classified patients according to highest achieved motor milestone and age at onset: SMA types 1c (head control or rolling over), 2a (sitting), and 2b (supported standing).^5^^,^^15^^,^^17^^,^^18^

### Ethics

All patients had previously given oral and written informed consent to participate in the longitudinal Dutch SMA registration study.^5^ The study was approved by the local medical ethics committee (METC No. 09307/NL29692.041.09) and was registered in the Dutch registry for clinical studies and trials (www.toetsingonline.nl).

### Procedures

Screening included blood and urine tests, and we performed an electrocardiogram before the start of treatment. After the first safety evaluation after 2 months of treatment, patients visited the outpatient clinic for follow-up every 8 months. Safety and efficacy analysis consisted of a systematic evaluation encompassing an interview, physical examination, blood and urine tests and electrocardiogram. All motor function tests were done by physiotherapists (BB, DW, TR, BV, SH, DD, JD) trained specifically to conduct RULM, ATEND, and hand strength tests.

We assessed *SMN1* and *SMN2* copy number status with SALSA Multiplex Ligation-dependent Probe Amplification (MLPA) kit P021 (version B1–01; MRC Holland). All MLPA reactions were conducted according to the manufacturer’s protocol (www.mlpa.com; www.mrcholland.com).

Preparation and administration of risdiplam were according to manufacturer’s instructions and label for patients over 20 kg and older than 2 years of age.^19^ Hence, all patients included in the study were treated with a daily dose of 5 mg risdiplam in oral suspension.

### Outcomes

We assessed motor function using the RULM. The RULM is a motor scale for assessing upper limb function with a maximum score of 37, designed for and validated in patients with SMA.^20^ Assessments were performed bilaterally and standardised. Floor effects are observed in patients with a baseline score below 10.^12^ We therefore also used the recently developed ATEND, for which initial validation has been performed,^13^^,^^21^^,^^22^ as a complementary outcome measure. It incorporates assessments of both axial and distal motor function and represents a promising scale to detect motor changes in this group of severely impaired individuals with SMA.^13^^,^^21^^,^^22^ Patients are examined in (semi-reclined) supported sitting position in their wheelchair using 14 items up to a maximum score of 46. Items are rated according to skill level, with maximum points varying by item (ranging from 2 to 4). A score of 0 is assigned when there is no movement of the tested muscle or the item cannot be completed. In addition, we assessed change in grip and pinch grip strength using hand-held dynamometry (MyoGrip and MyoPinch) in kilograms. The assessment was repeated (up to five times) until two measurements were within 10% of each other. After approximately 3 years of treatment, we asked patients to report change in their overall wellbeing and in their motor function using the PGIC scale once. To assess its correlation with motor function, the motor assessments closest in time were used. See Supplementary material III for a detailed description of these outcome measures.

We investigated changes in RULM score as our main outcome measure. We defined 1) improvement as gaining 2 or more points in total RULM scores, and 2) stabilisation and improvement as 0 or more points change in RULM, as defined by the secondary outcome of the pivotal risdiplam trial.^9^^,^^23^ Given the advanced disease severity in this population, we hypothesised that motor function would remain stable during treatment, as we considered acquisition of new motor skills and strength unlikely. We regarded stabilisation of motor function as a favourable outcome, since natural history studies of SMA have shown that deterioration of motor function is the rule rather than the exception and patients view stabilisation as a relevant outcome.^5^^,^^24^ To include multiple aspects of motor functionality and to address the problem of the floor effect of the RULM in a population with severely limited (arm) motor function, we assessed changes in total ATEND score as an additional outcome measure.^12^^,^^22^ We did not predefine response on the ATEND in the absence of studies that defined day-to-day variation but described changes in ATEND as increases or decreases relative to baseline. In addition, we assessed construct validity and responsiveness of the ATEND in patients with SMA type 1c and 2 who have limited motor function.

### Statistics

We used descriptive statistics to summarise baseline characteristics and Fisher’s exact test (categorical variables) and the Wilcoxon rank-sum test or Kruskal–Wallis test (continuous variables) to compare characteristics between subgroups and across follow-up visits. To compare (relative) motor function scores between baseline and follow-up visits, we used Wilcoxon Rank Sum test with continuity correction for pairwise comparison. In addition, paired t-tests were conducted to assess the robustness of the findings in the presence of tied values. We used linear mixed-effects models to model longitudinal trajectories of the change in motor function scores during follow-up. The dependent variable was the RULM score. We included time since start of treatment in years – both linear and quadratic terms – as fixed effects and subject-specific random intercepts and slopes for time. Models were estimated using maximum likelihood, which yields valid inferences under the missing at random assumption. No formal imputation was performed. To investigate the effect of different baseline characteristics (e.g., SMA type, *SMN2* copy number, baseline motor function score) on motor function score trajectories over time, we included interaction terms between both linear and quadratic time and each baseline characteristic. Significance of the interaction terms was based on a likelihood ratio test with 2 degrees of freedom. To address potential limitations in responsiveness at the lower end of the RULM scale, we performed subgroup analysis stratified by baseline RULM score (<5 versus ≥5). We analysed correlation between outcome measures using Spearman or Kendall Tau correlation tests as appropriate. We performed a post-hoc analysis to estimate the minimal clinically important difference (MCID) for the ATEND score via two methods. First, we performed an anchor-based method based on the mean change in ATEND score of self-reported improved patients. Second, we performed an anchor-based distribution model based on ROC analysis to estimate the optimal cut-off to discriminate between self-reported improved and non-improved patients.^25^^,^^26^ For these analyses, we defined improvement on the anchor (i.e., PGIC) as self-reported ‘minimally improved’ or more. The highest of the calculated MCID values was selected to apply the most conservative threshold for clinically meaningful change. We excluded four patients aged younger than 16 years from all analyses. All statistical analysis were done using R software (4.1.1. for macOS and RStudio version 2024.09.1 + 394, R foundation for Statistical Computing, Vienna, Austria). The significance level was set at p < 0.050.

### Role of the funding source

There was no funding source for this study.

## Results

We analysed 72 patients with SMA type 1c and SMA type 2. Apart from one patient, all patients were older than 18 years (range 16–52) at start of treatment. Patients’ characteristics are summarised in Table 1. Patients’ baseline motor function scores were lower and the need for invasive ventilation was higher in more severe disease, as expected (p ≤ 0.027, Supplementary material V: Supplementary Table S2). Baseline RULM and ATEND scores and pinch grip strength were higher in female patients than male patients (p ≤ 0.0059).

**Table 1:** Patients’ characteristics at start of treatment.

	Cohort (N = 72)
Sex female/male – n (%)^a^	44 (61)/28 (39)
SMA type – n (%)	
1c	8 (11)
2a	43 (60)
2b	21 (29)
*SMN2* copy number – n (%)	
2^b^	2 (3)
3	63 (88)
4	7 (10)
Median age at baseline (IQR) – years	29 (23–42)
Scoliosis/Scoliosis surgery – n (%)	72 (100)/66 (92)
Ventilation non-invasive/invasive – n (%)	27 (38)/10 (14)
Gastrostomy – n (%)	13 (18)
Median motor measures – (IQR)	
RULM score (0–37)	6 (2–15)
Score 0–n (%)	16 (22)
Entry item A^c^	
0	1 (1)
1	36 (50)
2	5 (7)
3	29 (40)
4	1 (1)
ATEND score (0–46)^d^	24 (20–32)
Grip strength^e^	1.00 (0.45–1.89)
Pinch grip strength^e^	0.432 (0.228–0.654)
Maximal inspiratory pressure^f^	50 (37–67)
Maximal expiratory pressure^f^	27 (23–38)

Percentages may not sum to 100 due to rounding.

ATEND = Adapted Test of Neuromuscular Disorders. RULM = Revised Upper Limb Module. SMA = spinal muscular atrophy.

a Assigned at birth.

b In both patients with 2 copies a heterozygous c.859G>C variant was present in the *SMN2* gene.

c No patients scored 5 or 6 for Entry Item A of the RULM. Entry item A evaluates global hand and arm function with 6 response options, ranging from no hand and arm function (score 0), the ability to raise a cup with 200 g to their mouth using one or both hands (score 3), and the ability to abduct both arms simultaneously (elbows in extension) in a full circle until they meet above the head (score 6).

d n = 67.

e Grip strength (n = 65) and pinch grip strength (n = 71) are measured and presented in kilograms (kg).

f Maximal inspiratory pressure (n = 66) and expiratory pressure (n = 55) are measured and presented in centimetres of water (cmH_2_O).

We treated patients for a median period of 35 months (interquartile range (IQR) 34–37): median treatment duration was 9 months (IQR 9–10; n = 69) at visit 2 (hereafter 9 months), 19 months (IQR 19–20; n = 66) at visit 3 (hereafter 19 months), 28 months (IQR 27–29; n = 62) at visit 4 (hereafter 28 months), and 36 months (IQR 35–37; n = 60) at visit 5 (hereafter 36 months). Patients’ characteristics remained comparable to baseline at all follow-up visits. We included a study flow diagram detailing inclusion, follow-up, and pairwise analyses in Supplementary material VI: Supplementary Fig. S1.

Five patients intensified respiratory support (starting non-invasive n = 2; changing from non-invasive to tracheostomy n = 3) during follow up but were already in a respiratory decline before start of treatment. Four patients got a gastrostomy during follow-up.

**Fig. 1:**
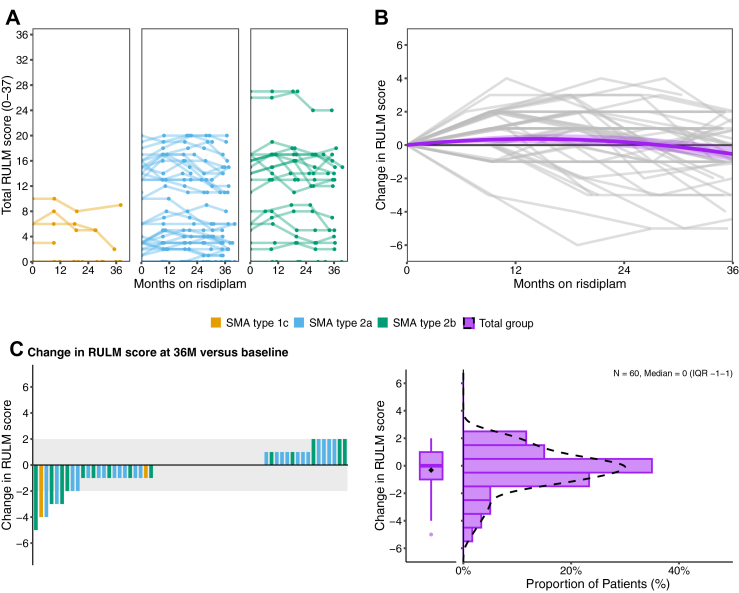
**RULM scores of patients during treatment with risdiplam. (A,B)** The spaghetti plot represents individual patient trajectories of the total RULM score over time stratified for SMA type (indicated by different colours) and delta RULM score (grey). **(A)** The dots represent the different timepoints for assessment during follow-up. The individual trajectories of RULM score during risdiplam illustrate the differences in time to observed change. **(B)** The purple line and purple shading reflect an estimation of the average change in RULM and the 95% CI, respectively. **(C)** Individual RULM score changes at 36 months are depicted as bars; 21 patients without changes at 36 months compared to baseline are reflected by the straight line. The grey area represents changes within ±2 points, which is considered day to day variation.^27^ The histogram shows the proportion of patients (%) with specific score changes at 36 months versus baseline. On the left, mean (diamond), median (line), IQR (box), 1.5 × IQR (whiskers), and outliers (dots) are indicated. The dashed line shows the kernel density. See Supplementary Fig. S2 for previous visits. M = months. RULM = Revised Upper Limb Module. SMA = spinal muscular atrophy.

Median baseline RULM score was 6 (IQR 2–15). Sixteen (22%) patients had a RULM score of 0 and 40 (56%) of <10 at baseline. Median RULM score initially improved at 9 months (p = 0.00064) to 8 (IQR 2–16) from 6 (IQR 2–15) at baseline and stabilised over time to 6 (2–14) at 36 months (Fig. 1A and B and Supplementary material VII: Supplementary Table S3). Including or excluding patients with baseline RULM of 0 did not alter results or interpretation of significance (p = 0.00064 at 9 months and p = 0.029 at 19 months). Baseline motor function scores and *SMN2* copy number were associated with differences in the shape of RULM score trajectories over time (p ≤ 0.018 and p = 0.046, respectively). Although statistically significant, the magnitude of these differences appeared modest, and their clinical relevance is probably limited. Other patient characteristics were not associated with higher RULM scores.

During treatment with risdiplam, 26 of 60 (43%) patients stabilised or improved in RULM score (Fig. 1C and Supplementary material VIII: Supplementary Fig. S2). Although total RULM scores of 11 (18%) patients (n = 3 SMA type 1c, n = 7 SMA type 2a, and n = 1 SMA type 2b) remained 0 from baseline to 36 months, ATEND scores of 10 of these patients increased. The ATEND score of one patient decreased by 1 point at 28 months. Scores of two patients without ATEND baseline measurement gained a point (n = 1) or remained unchanged (n = 1) from 9 months to 36 months.

Subgroup analysis of patients with baseline RULM score of 5 or more points (n = 41) demonstrated stabilisation of median RULM score, remaining at 14 (IQR 10–16) at baseline to 14 (IQR 8–15) at 36 months (Supplementary material IX: Supplementary Fig. S3). The distribution of patients (n = 34) gaining points, stabilising, or losing points at 36 months differed between those with baseline scores <5 and ≥5 (p = 0.0034). A higher proportion of patients in the group with baseline RULM score ≥5 both improved and declined in points compared to those with a baseline score below 5 (Supplementary Fig. S3); however, when excluding patients (n = 11) with stable RULM score of 0 the proportions did not differ (p = 0.47). Of the patients with baseline RULM score < 5 (n = 26), none lost ≥2 points, five lost 1 point, eight remained stable (≥0 points), and two gained ≥2 points.

**Fig. 2:**
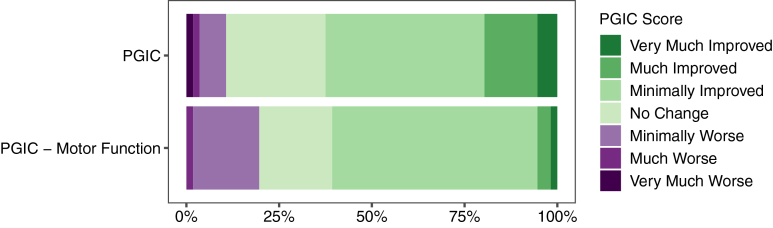
**Change in PGIC after more than 3 years of risdiplam treatment.** Patients (n = 56) reported their PGIC after >3 years of treatment, six patients’ PGIC were missing due to discontinued treatment (n = 1), choice (n = 1), unknown (n = 4). **PGIC:** Very much worse (n = 1, 2%), Much worse (n = 1, 2%), Minimally worse (n = 4, 7%), No change (n = 15, 27%), Minimally improved (n = 24, 43%), Much improved (n = 8, 14%), Very much improved (n = 3, 5%). **PGIC—Motor Function:** Very much worse (n = 0, 0%), Much worse (n = 1, 2%), Minimally worse (n = 10, 18%), No change (n = 11, 20%), Minimally improved (n = 31, 55%), Much improved (n = 2, 4%), Very much improved (n = 1, 2%). PGIC=Patient Global Impression of Change.

Fifty-six patients reported (when asked) their impression of change of motor function after a median of 42 months (IQR 37–43) of treatment (Fig. 2). Changes in RULM partially aligned with reported change after treatment on the PGIC in motor function (n = 39, 70%) (Supplementary material X: Supplementary Fig. S4A,C): 11 (20%) patients, however, reported improvement or stability while objectively declining in RULM score. In other words, 67% (n = 11/16) of patients declining in RULM score reported improved motor function. Six (10%) patients reported deterioration while their scores stabilised or improved.

Motor function changes not captured by RULM score included head control, trunk strength, fine motor skills, and ease of performing daily activities. Apart from motor function improvement, patient comments included improved lung function (n = 17), bulbar function (n = 9), endurance or energy (n = 7), mental state (n = 2), disease recovery (n = 2), and stool (n = 2). Apart from motor function decline, one patient reported a decline in endurance.

**Fig. 3:**
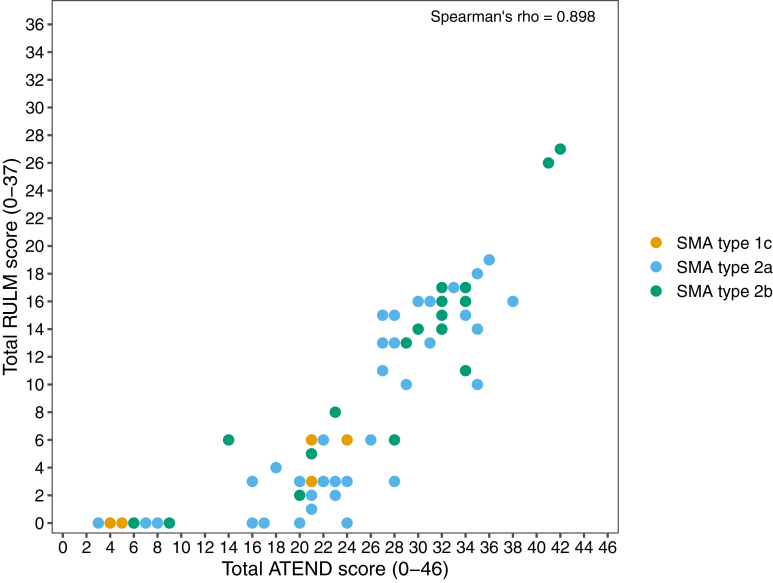
**Correlation of RULM score and ATEND score.** Dots represent individual patients (color-coded by SMA type) with available baseline score of RULM and ATEND (n = 67). ATEND = Adapted Test of Neuromuscular Diseases. RULM = Revised Upper Limb Module. SMA = spinal muscular atrophy.

The RULM and ATEND score strongly correlated at baseline and longitudinally (Spearman ρ = 0.90; p < 0.0001; Fig. 3 and Supplementary material XI: Supplementary Fig. S5). We observed changes in the ATEND scores of patients at the lower range of the RULM.

**Fig. 4:**
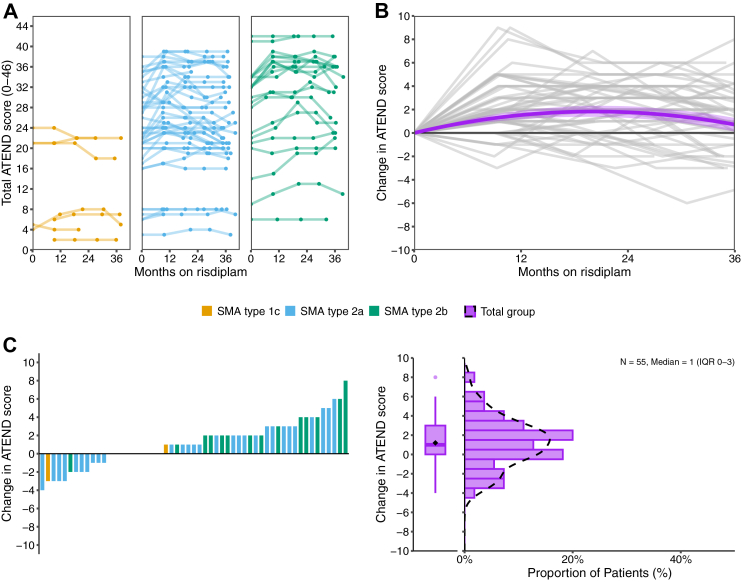
**ATEND scores of patients during risdiplam treatment. (A,B)** The spaghetti plot represents individual patient trajectories of the total ATEND score over time stratified for SMA type (indicated by different colours) or delta ATEND score (grey). **(A)** The dots represent the different timepoints for assessment during follow-up. Patients with milder SMA types score higher at baseline but with a broad range of scores even within the same SMA type. Individual trajectories of ATEND score during risdiplam illustrate the differences in time to observed change. **(B)** The purple line and purple shading reflect an estimation of the average change in ATEND and the 95% CI. **(C)** Individual ATEND score changes at 36 months are depicted as bars; 9 patients without changes at 36 months compared to baseline are reflected by the straight line. The histogram shows the proportion of patients (%) with specific score changes at 36 months versus baseline. On the left, mean (diamond), median (line), IQR (box), 1.5 × IQR (whiskers), and outliers (dots) are indicated. The dashed line shows the kernel density. See Supplementary Fig. S6 for previous visits. ATEND = Adapted Test of Neuromuscular Diseases. M = months. SMA = spinal muscular atrophy.

Median baseline ATEND score was 24 (IQR 20–32). Median ATEND score increased to 27 (IQR 20–35; p < 0.0001) during follow-up and stabilised over time to 25 (IQR 20–33, p = 0.0023; Fig. 4A,B and Supplementary Table S3). SMA type and sex were associated with differences in the shape of ATEND score trajectories during treatment (p = 0.0017 and p = 0.015, respectively). While these differences were statistically significant, the extent appeared modest, and their clinical relevance is probably limited. Other patient characteristics including baseline ATEND score were not associated with improvement in ATEND score during treatment.

The proportion of patients (per SMA type) changing in ATEND score during risdiplam at 9, 19, and 28 months are shown in Supplementary material XII: Supplementary Fig. S6. At 36 months, the change in ATEND score relative to baseline of 33 (60%) patients exceeded the calculated MCID of +1 point (Fig. 4C and Supplementary material XIII), suggesting that, for those patients, the observed change was clinically meaningful. Improvement or stability was noted in both the upper limb function (i.e., item 1, 4, 8, 9, 12–14; n = 38; 69%) and axial function (i.e., item 5–7, 11; n = 39; 71%) at 36 months.

We found that 54, 47, 39, and 28 (84, 77, 67, and 51%) patients were stable or gained points and 2, 1, 6, and 6 (3, 2, 10, and 11%) patients lost points on both RULM and ATEND at 9, 19, 28, and 36 months, respectively (Supplementary Fig. S5). All 11 patients who were not able to perform the RULM (i.e., RULM score of 0 across all visits) showed stable or increased ATEND scores during treatment, except for one patient (−1 point on the ATEND score). Of the patients who deteriorated more than 2 points on RULM or ATEND score (n = 11) at one point during follow up, the scores of two patients were influenced by severe systemic illness during or before assessment (e.g., systemic infection ± intensive care unit admission), and most points were regained at the follow up visits.

Patients had problems with performing the grip and pinch test reliably, which led to grip or pinch grip strength being available in only 66% (n = 41) and 53% (n = 33), respectively, after 28 months of treatment. We, therefore, excluded the grip and pinch grip strength measure in the treatment efficacy assessment to limit possible introduction of bias. For clarity and to prevent reporting bias, descriptive statistics of grip and pinch grip strength during follow up are summarised in Supplementary material XIV: Supplementary Table S4.

Six patients discontinued treatment after a median of 23 months (IQR 20–29), either due to adverse events (all gastrointestinal complaints), the physical and logistical burden of hospital visits exceeding their perceived benefit of treatment, or a combination of both (Supplementary Fig. S1). One patient discontinued treatment due to perceived deterioration with a loss of more than 2 points in RULM score, but a 3-point gain in ATEND score. Five patients died during the observed treatment period (SMA type 1c and 2a; causes of death were pneumonia in two, post-operative complication after nephrectomy, sepsis secondary to acute cholecystitis, and paracetamol-induced liver failure), all unrelated to risdiplam treatment. All adverse events (n = 303 in 67/72 patients) during risdiplam are summarised in the Supplementary material XV: Supplementary Table S5. The most common adverse event was respiratory tract infection (n = 85, 45 patients), followed by changed stool (n = 20, 16 patients) and dermatological complaints (n = 20, 16 patients), such as rash or increased sun sensitivity.

## Discussion

In our study, we observed improved or stabilised RULM scores in 26 (43%) adult patients with SMA type 1c and 2, despite longstanding disease and severe motor impairment, after a median of 36 months of treatment with risdiplam. In addition, ATEND scores improved or were stable in at least 33 (60%) patients, including those with RULM scores of 0. After improvement, motor scores plateaued at a new level, similar to observations in much younger patients during DMT.^28^ Additionally, up to 89% of patients self-reported improvement or stabilisation of motor function and overall wellbeing during the treatment period. Thus, this population-based study provides the first real-world evidence of motor function changes during treatment with risdiplam in treatment-naive adult patients with severe SMA without alternative therapeutic options.

The observed stabilisation in RULM in our study cohort is different from the progressive deterioration shown in natural history studies of SMA type 1c and 2, which might suggest treatment effect of risdiplam.^23^^,^^29^, ^30^, ^31^ This is supported by natural history data of RULM, which describe an average deterioration of 1.67 (SD 2.33) points in (young) adults with SMA type 2 (n = 18; age range 15–50 years) after 2 years.^23^ Moreover, stability in motor function aligns with patients’ own perspective on treatment success and underscores its clinical relevance.^32^ The initial improvement followed by stabilisation has been reported in patients with SMA after prolonged treatment with DMT.^28^^,^^33^ We hypothesise that the initial improvement may reflect the activation of hibernating motor units,^34^^,^^35^ whereas the following plateau may represent the limitation in the number of motor units that can be (persistently) maintained after treatment with SMN-targeting therapy in patients with symptomatic SMA.

RCTs are increasingly being complemented by real-world data,^33^^,^^36^, ^37^, ^38^, ^39^, ^40^ which often include highly heterogeneous populations (e.g., SMA types 1–4) and frequently report combined analyses across disease subtypes. This heterogeneity may result in overall motor function trajectories that do not accurately reflect those of more severely affected patients. This could explain why Keritam and colleagues reported sustained improvements in RULM and HFMSE, in contrast to our findings. In comparison, in a cohort of non-ambulatory patients (SMA types 1–3), Parmova and colleagues observed an initial improvement followed by stabilisation, which is consistent with our results. It remains important to assess SMA across all subtypes and functional stages in order to fully understand treatment effects across the spectrum.

Although our study findings are supported by other observations in adults with severe SMA,^37^^,^^38^ we likely underestimated the effects of risdiplam by primarily considering RULM scores for three reasons. First, we used a strict definition for stability as no change in score and improvement as 2 or more points due to limited natural history data. This was substantiated by the fact that 67% of patients who did not meet our definitions nonetheless self-reported that they had improved or were stable. We used this definition of the primary outcome measure from the pivotal risdiplam trial to ensure methodological consistency and facilitate comparison with existing data. In addition, the widely adopted definition of treatment response as >2 points in clinical efficacy studies is based on natural history studies of SMA type 2 and 3 in younger patients and is, therefore, not suitable for our study population.^23^ Second, RULM is a relatively insensitive measure in this population. While RULM is a reliable and validated scale for assessment of upper limb function of patients with SMA type 2 and 3, its clinical applicability is constrained by floor effects in patients with RULM scores below 10.^12^ In our population, 56% of patients scored <10 at baseline, of whom 11 (out of 40) started at score 0, which did not improve during follow up, signalling that RULM may be less responsive in this population. Third, our focus on motor function assessments in patients with severe disability may not fully reflect treatment effects or their clinical relevance. This is further supported by the proportion of self-reported overall improvement or stability exceeding that of motor function assessment.

Accordingly, reliance on RULM scores alone is insufficient in our population. Our data of construct validity and responsiveness of the ATEND show that it is useful to assess motor function changes in weaker patients with SMA. RULM and ATEND score strongly correlated and the ATEND was of added value by capturing motor function changes of patients whose RULM scores did not change. The RULM was designed for assessing upper limb function in non-ambulant patients, but the ATEND is useful when RULM scores are <10, i.e., within the floor effect.^12^ The inclusion of items pertinent to axial function and the incorporation of gravity-eliminated testing, enhance the value of the ATEND in monitoring motor function changes even in patients with severe impairments.

Our data suggests that for 60% of patients the observed motor function change in ATEND score was clinically meaningful, based on the estimated MCID of +1.0, calculated via exploratory anchor-based analyses.^25^^,^^26^ We were not able to report an MCID for RULM due to the previously mentioned insensitivity of the RULM in our study population, resulting in lack of association between our anchor (i.e., PGIC) and change in RULM score. This discordance between RULM responder status and patients’ experience of treatment effect in the SMA population has also been reported after nusinersen treatment.^41^ However, in part, the discordance between motor function scores and PROMs may be attributed to the inherent potential for recall bias in a scale evaluating change in combination with longer treatment duration and the time lag between assessments. In addition, patients’ expectation or confirmation biases due to unblinded motor assessments may have influenced the results.

In our study population, the technical feasibility of hand strength assessment by a hand-held dynamometer depended heavily on the positioning, causing results to be highly variable. This has been observed previously by Iterbeke and colleagues, who reported difficulties in measuring hand grip strength in the majority of their population.^39^

The observed insensitivity (e.g., RULM) and infeasibility (e.g., hand-held dynamometer) of current motor function outcome measures do not only underscore the need for novel outcome measures, but also the importance of inclusion of PROMs (e.g., PGIC). These provide insight into the patients’ perspective, capture changes that may not be reflected by motor function scales and allow evaluation of clinical meaningfulness. The large majority (89%) of patients self-reported improvement or stability on the PGIC during risdiplam treatment, which is in line with previous studies that used PROMs.^37^ Interestingly, confirming the additive value of PROMs to motor function scales, multiple patients reported improvements on various aspects other than changes in upper limb or axial function (i.e., lung function, bulbar function, endurance or energy, disease recovery, stool and mental state). To monitor the full range of treatment response in the future, we should consider assessing PROMs at baseline and across all follow-ups, in addition to relevant motor function scales and electrophysiology (e.g., CMAP scan) or other biomarkers.

This study has several important strengths. The population-based approach of our study enhanced generalisability and provided real-world data on motor function changes during risdiplam in severely affected adult SMA patients. Moreover, our data showed stabilisation and improvement of motor function scores during risdiplam treatment in SMA type 1 and 2 despite their advanced disease stage. Since changes in motor function scores greater than day-to-day variation are uncommon and gradual decline in motor function characterises the natural history of SMA, this might suggest that SMN upregulation remains an effective therapeutic strategy for SMA even in the severe end of the spectrum. We acknowledge that our data require confirmation in representative populations. To our knowledge, this is the largest cohort study to date on motor function change during risdiplam in adults with SMA type 1 and 2. The prolonged follow-up period of more than 3 years allowed us to compare motor function changes with the natural history of SMA. Furthermore, we included the ATEND and provided first evidence for construct validity and responsiveness. We also used patient-reported PGIC to assess treatment response. The interpretation of the PROM may be limited by placebo effects in the context of a highly anticipated treatment, patients’ expectation and confirmation biases from unblinded motor assessments, and recall bias following prolonged treatment in the absence of longitudinal evaluation. These factors should be considered when interpretating the results. Another limitation is the absence of a control group or pretreatment RULM or ATEND measurements as comparator to assess the individual changes of progression before and after start of treatment with risdiplam. However, our nationwide observational cohort study is an appropriate design to provide real-world data on changes during risdiplam, as inclusion of a treatment-naïve control group would not be ethically justifiable.

In conclusion, motor function changes in adults with SMA type 1c and 2 during risdiplam treatment differ from the natural progressive disease course even after longer disease duration. This suggests that risdiplam could benefit adults with SMA with severe motor restrictions due to longstanding disease. Patient-reported stability and improvement further highlight the clinical relevance of the observed changes during follow-up. Moreover, the ATEND represents an important additional scale to the RULM when assessing motor function change in severely affected patients with SMA.

## Contributors

FA, RPAE, RIW, and WLP designed the study. LMV, FA, DRW, BMHEAV, SMJH, TJR, BB, IC collected data. LMV and FA accessed and verified the underlying data. Data analyses were conducted by LMV and RPAE and reviewed or interpretated by all authors. LMV, RIW and WLP drafted the first manuscript. All authors critically reviewed and revised the manuscript and figures and approved the final version of the manuscript. All authors had full access to all the data and have the right to publish independently of any sponsor.

## Data sharing statement

The anonymised data supporting the findings of this study are available from the corresponding author upon reasonable request.

## Declaration of interests

IC consults ad hoc for Novartis (all fees to employer). BB is on the scientific advisory board of Scholar Rock and receives research support from Prinses Beatrix Spierfonds, Stichting Spieren voor Spieren, Piet Poortman Fonds and SIA-RAAK. WLP is on the scientific advisory board of SMA Europe and receives research support from Prinses Beatrix Spierfonds, Stichting Spieren voor Spieren, and Vriendenloterij. BB and WLP consult (ad hoc) for Biogen, Novartis, Scholar Rock and Roche, and WLP also for Biohaven and NMD Pharma (all fees to employer). RW receives research support from Prinses Beatrix Spierfonds, Stichting Spieren voor Spieren and the Dutch Research Council (NWO), and consults ad hoc for Biogen and Novartis (all fees to employer). All other authors have no conflict of interests to declare.

## Appendix A Supplementary data

Supplementary materials I–XV
