# Supplementary material for: Motor function score changes in severe 5q spinal muscular atrophy during risdiplam treatment: an observational longitudinal nationwide cohort study
Source: eClinicalMedicine. 2026 Jun 30;97:104008. doi: 10.1016/j.eclinm.2026.104008 (PMC13420608; doi:10.1016/j.eclinm.2026.104008)
Supplement: Supplementary materials I–XV [file mmc1.pdf]

Supplementary Material - ‘Motor function score changes in severe 5q spinal muscular atrophy during risdiplam treatment’

Table of contents **page**

I. Supplementary table S1: Summary of the literature on risdiplam efficacy in treatment naïve patients	2, 3
II. Search strategies	3
III. Detailed description of motor function outcomes	4
IV. Patient Global Impression of Change (PGIC) Questionnaire (Dutch)	5
V. Supplementary table S2: Patients’ characteristics	6
VI. Supplementary figure S1: Study flow diagram	7
VII. Supplementary table S3: Changes is motor function scores	8
VIII. Supplementary figure S2: Changes in RULM score at 9M, 19M, 28M	9
IX. Supplementary figure S3: Subgroup analysis (baseline RULM ≥5)	10
X. Supplementary figure S4: Association between PGIC and ATEND or RULM	11
XI. Supplementary figure S5: Correlation ATEND and RULM over time	12
XII. Supplementary figure S6: Changes in ATEND score at 9M, 19M, 28M	13
XIII. MCID estimation of the ATEND	14
XIV. Supplementary table S4: Hand grip strength	15
XV. Supplementary table S5: Adverse events	16
REFERENCES	17

I. Supplementary table S1: Summary of the literature on risdiplam efficacy in treatment naïve patients

Supplementary table S1. Chronological overview of studies on risdiplam efficacy with RULM data where available

Author	Year	SMA type (n, %)	n	Age at inclusion	Adults treated, n (%)	Treatment efficacy assessment, months	Outcome measures	RULM baseline	RULM outcome	RULM mean difference
Baranello et al. (FIREFISH-I)[1]	2021	1	21	3–7 months	0 (0)	12	BSID-III, HINE-2, CHOP-INTEND	NA	NA	NA
Masson et al. (FIREFISH-II)[2]*	2022	1	41	1–7 months	0 (0)	12, 24	BSID-III, HINE-2, CHOP-INTEND	NA	NA	NA
McCluskey et al.[3]	2023	2	6	26–44 years	6 (100)	9	RULM, FEV1, FVC, QOLM, EK2, ESS	8.6†	11.8†	3·2 (–0·13–6·53)
Mercuri et al. (SUNFISH-I)[4]	2023	2 (37, 73%),<br>3 (14, 27%, non-ambulatory)	51	2–24 years	4 (8)	12, 24	MFM-32, RULM, HFMSE, FVC, FEV1, PCF, SNIP	··	··	All: 1·72 (0·90–2·54), 2·48 (1·52–3·44)<br>12–25 years: 1·05 (0·18–1·92), 1·74 (0·59–2·88)
Oskoui et al. (SUNFISH-II)[5]**	2023	2 (81, 70%),<br>3 (34, 30%, non-ambulatory)	120	2–25 years	10 (8)	12, 18, 24	MFM-32, RULM, HFMSE, SNIP, FVC, FEV1, MIP, MEP, SMAIS-ULM	··	··	1·9 (1·2–2·6), 2·1 (1·3–2·8), 2·8 (1·9–3·6); Placebo switched at 12M: 0·9 (0·1–1·6)
Sitas et al.[6]	2024	2 (15, 48%),<br>3 (16, 51,6%)	31	18–65 years	31 (100)	16 or 31	RHS, RULM, 6MWT, INQoL, PROM-JFLS	··	··	SMA type 2: 0 (1), 0·33 (1·3); SMA type 3: 1·63 (3·6)
Brakemeier et al.[7]	2024	2 (24, 96%),<br>3 (1, 4%)	25	19–58 years	25 (100)	12	ALSFRS-R, SSQ, HFMSE, RULM	8·84 (5·11)	9·16 (5·73)	0·32†
Iterbeke and Claeys[8]	2025	2 (8, 44%), 3 (9, 50%), 4 (1, 6%)	18	Median of 38·6 years	18 (100)	12, 24	MMT, Hand Strength, RULM, MFM-32, FVC, PEF, NdSSS, FOIS, SSQ, FSS, SMAIS, SF-36	17·0 (11·6)	12M: 18·0 (11·3), 24M: 18·6 (10·8)	12M: 1·0 (0·2–1·8), 24M: 1·0 (−0·2–2·2)
Keritam et al.[9]	2025	1 (2, 4%), 2 (23, 40%), 3 27, 47%), 4 (4, 7%), unknown (1, 2%)	57	Median of 35·7 years (IQR 28·8–43·4)	·· ‡	3–<6, 6–<12, 12–<18, ≥18	RULM, 6MWT, HFMSE, ALSFRS-R	16·9 (12·7)	··	12–<18M: 2·51 (1·48–3·55), ≥18M: 2·76 (1·51–3·94)
Parmova et al.[10]§	2026	1 (4, 9%), 2 (28, 64%), 3 (12, 27%)	44	18-59 years	44 (100)	6, 12, 24, 36	HFMSE, RULM	··	··	··

Legend

Studies were included when:

- Patients were treatment-naïve before starting risdiplam; were not previously treated with other SMN-targeting therapies for SMA.
- Follow-up duration was more than 6 months.
- Data on treatment efficacy was available at fixed follow-up time-points.
- Published in English.

We excluded pre-clinical studies, (systematic) reviews or single case reports.
We searched PubMed and Embase up to April 1st, 2026**,** combining 'spinal muscular atrophy' (or 'SMA') and 'risdiplam' (or 'Evrysdi' or 'RG7916' or 'RO7034067') (see **Supplementary Material II** for full search strategies).
All RULM scores are reported in mean (95%-CI) except for Sitas et al., Brakemeier et al., and Parmova et al. who reported scores in mean (SD), and Iterbeke et al. who reported total scores in mean (SD) and difference of scores in mean (95%-CI). * A 12-month interim analysis has been published in 2021 by Darras et al.[11] ** A 12-month interim analysis has been published in 2022 by Mercuri et al.[12] † No reference range available, however change was reported to be significant. ‡ All patients were 16 years of age or older. § We reported the variables of the included treatment naïve subgroup (n=44 of 59 patients). Total motor function scores or compared to baseline are not separately available for treatment naïve patients.
6-MWT 6-Minute Walk Test; *ALSFRS-R* Amyotrophic Lateral Sclerosis Functional Rating Scale Revised; *BSID-III* Bayley Scales of Infant and Toddler Development, third edition; *EK-2* Egen Klassifikation 2; *ESS* Epworth Sleepiness Scale; *FEV1* Forced Expiratory Volume in One Second; *FOIS* Functional Oral Intake Scale; *FSS* Fatigue Severity Scale; *FVC* Forced Vital Capacity; *HFMSE* Hammersmith Functional Motor Scale – Extended; *HINE-2* Hammersmith Infant Neurological Examination, Module 2; *INQoL* Individualised Neuromuscular Quality of Life questionnaire; *M* Months; *MEP* Maximal Expiratory Pressure; *MFM-32* Motor Function Measure for Neuromuscular Disease 32; *MIP* Maximal Inspiratory Pressure; *NA* Not Applicable; *NdSSS* Neuromuscular Disease Swallowing Status Scale; *PCF* Peak Cough Flow; *PEF* Peak Expiratory Flow; *PROM-JFLS* Patient Reported Outcome Measure – Jaw Functional Limitation Scale; *QOLM* Quality of Life Measure for People with Slowly Progressive and Genetic Neuromuscular Disease; *RHS* Revised Hammersmith Scale; *RULM* Revised Upper Limb Module; *SMA* Spinal Muscular Atrophy; *SMAIS-ULM* SMA Independence Scale – Upper Limb Module; *SNIP* Sniff Nasal Inspiratory Pressure; *SSQ* Sydney Swallow Questionnaire

II. Search strategies

PubMed search strategy (searched November 26th, 2025): ("Spinal Muscular Atrophies of Childhood"[Mesh] OR "SMA"[Title/Abstract] OR "spinal muscular atroph*"[Title/Abstract]) AND ("risdiplam"[Title/Abstract] OR "evrysdi*"[Title/Abstract] OR "RG7916"[Title/Abstract] OR "RO7034067"[Title/Abstract])

Embase (searched June 11th, 2025): ('hereditary spinal muscular atrophy'/exp OR 'SMA':ti,ab,kw OR 'spinal muscular atroph*':ti,ab,kw) AND ('risdiplam'/exp OR 'risdiplam':ti,ab,kw OR 'evrysdi*':ti,ab,kw OR 'RG*7916':ti,ab,kw OR 'RO*7034067':ti,ab,kw)

III. Detailed description of motor function outcomes

The RULM consists of 20 items (A – T) that reflect proximal and distal upper limb function and has a maximum of 37 points. The RULM entry item (A) is scored independently and not included in the total score. It evaluates global hand and arm function with 6 response options, ranging from no hand and arm function (score 0), the ability to raise a cup with 200g to their mouth using one or both hands (score 3), and the ability to abduct both arms simultaneously (elbows in extension) in a full circle until they meet above the head (score 6). All other items are scored 0 to 2 based on the ability to complete the task (2 points) - with compensation (1 point) - or not at all (0 points). RULM total scores are based on the dominant or preferred side.

The ATEND is specifically designed (but not yet validated) for patients with more severe weakness, who use (electric) wheelchairs.[13–15] Patients are examined in (semi-reclined) supported sitting position in their wheelchair on 14 items (1 – 14) that encompass axial (item 5, 6, 7, 11), distal and proximal muscle function tests (upper limb: item 1, 4, 8, 9, 12, 13, 14; lower limb: item 2 and 10). The maximum ATEND score is 46: items are scored based on skill level from 0 to 2, 3, or 4. A score of 0 is assigned when there is no movement of the tested muscle or the item cannot be completed.

We assessed patients grip and pinch grip strength in kilograms with the MyoGrip and MyoPinch dynamometer. This method has been validated previously and has been used in studies assessing treatment efficacy in patients with SMA.[16–18]

The Patient Global Impression of Change (PGIC) is derived from the caregiver impression of change.[19] Patients were asked to answer on a 7-point scale, ranging from 'very much worse' to 'very much improved' (Supplementary Material IV). It has been validated in other (neurological) disorders and previously used in SMA research.[20–22]

IV. Patient Global Impression of Change (PGIC) Questionnaire (Dutch)

Als je nu kijkt naar hoe het met jou gaat, voel jij je dan beter of slechter dan <u>voor</u> de behandeling met risdiplam?

[ ] Zeer veel slechter

[ ] Veel slechter

[ ] Slechter

[ ] Hetzelfde

[ ] Beter

[ ] Veel beter

[ ] Zeer veel beter

Als je nu kijkt naar jouw manier van bewegen, is dit dan beter of slechter dan <u>voor</u> de behandeling met risdiplam?

[ ] Zeer veel slechter

[ ] Veel slechter

[ ] Slechter

[ ] Hetzelfde

[ ] Beter

[ ] Veel beter

[ ] Zeer veel beter

Beschrijf hier eventueel specifieke voor- of achteruitgang sinds de behandeling met risdiplam:

V. Supplementary table S2: Patients' characteristics

Supplementary table S2. Patients' characteristics at start of treatment

	SMA type 1c (N=8)	SMA type 2a (N=43)	SMA type 2b (N=21)	p-value
**Sex female / male – n (%)**	6 (100) / 0 (0)	28 (62) / 17 (38)	13 (61) / 8 (38)	ns
***SMN2* copy number – n (%)**				ns
**2**	0 (0)	1 (2)*	1 (5)*	
**3**	8 (100)	39 (91)	16 (76)	
**4**	0 (0)	3 (7)	4 (19)	
**Age at baseline in years**	44 (30–49)	26 (23–39)	34 (26–43)	ns
**Scoliosis surgery – n (%)**	7 (88)	40 (93)	19 (90)	ns
**Non-invasive ventilation – n (%)**	4 (50)	18 (42)	5 (24)	ns
**Invasive ventilation – n (%)**	4 (50)	5 (12)	1 (5)	0·027
**Gastrostomy – n (%)**	5 (63)	6 (14)	2 (10)	0·014
**RULM score (0 – 37)**	2 (0–6)	4 (1–14)	14 (6–16)	0·0088
**Score 0 – n (%)**	4 (50)	10 (23)	2 (10)	ns§
**ATEND score (0 – 46)†**	21 (5–21)	24 (20–30)	31 (22–34)	0·028
**Grip strength‡**	0·46 (0·37–0·74)	1·00 (0·33–1·79)	1·83 (0·92–2·67)	0·0091
**Pinch grip strength‡**	0·224 (0·124–0·576)	0·354 (0·204–0·618)	0·607 (0·432–0·916)	0·0088
**Maximal inspiratory pressure¥**	21 (17–24)	50 (39–64)	66 (43–85)	0·00036
**Maximal expiratory pressure¥**	12 (6–16)	27 (23–36)	37 (25–45)	0·00059

All data presented by median (IQR) unless otherwise stated
* In both patients with 2 copies a heterozygous c.859G>C variant was present in the *SMN2* gene.
§ SMA type 1c and 2b differ (p=0·033).

† n=67

‡ Grip strength (n=65) and pinch grip strength (n=71) are measured and presented in kilograms (kg).

¥ Maximal inspiratory pressure (n=66) and expiratory pressure (n=55) are measured and presented in centimetres of water (cmH2O).

ATEND Adapted Test of Neuromuscular Disorders; *IQR* Interquartile range; *RULM* Revised Upper Limb Module; *SMA* spinal muscular atrophy; *SMN2* survival motor neuron 2; *n* number; *ns* non-significant

VI. Supplementary figure S1: Study flow diagram

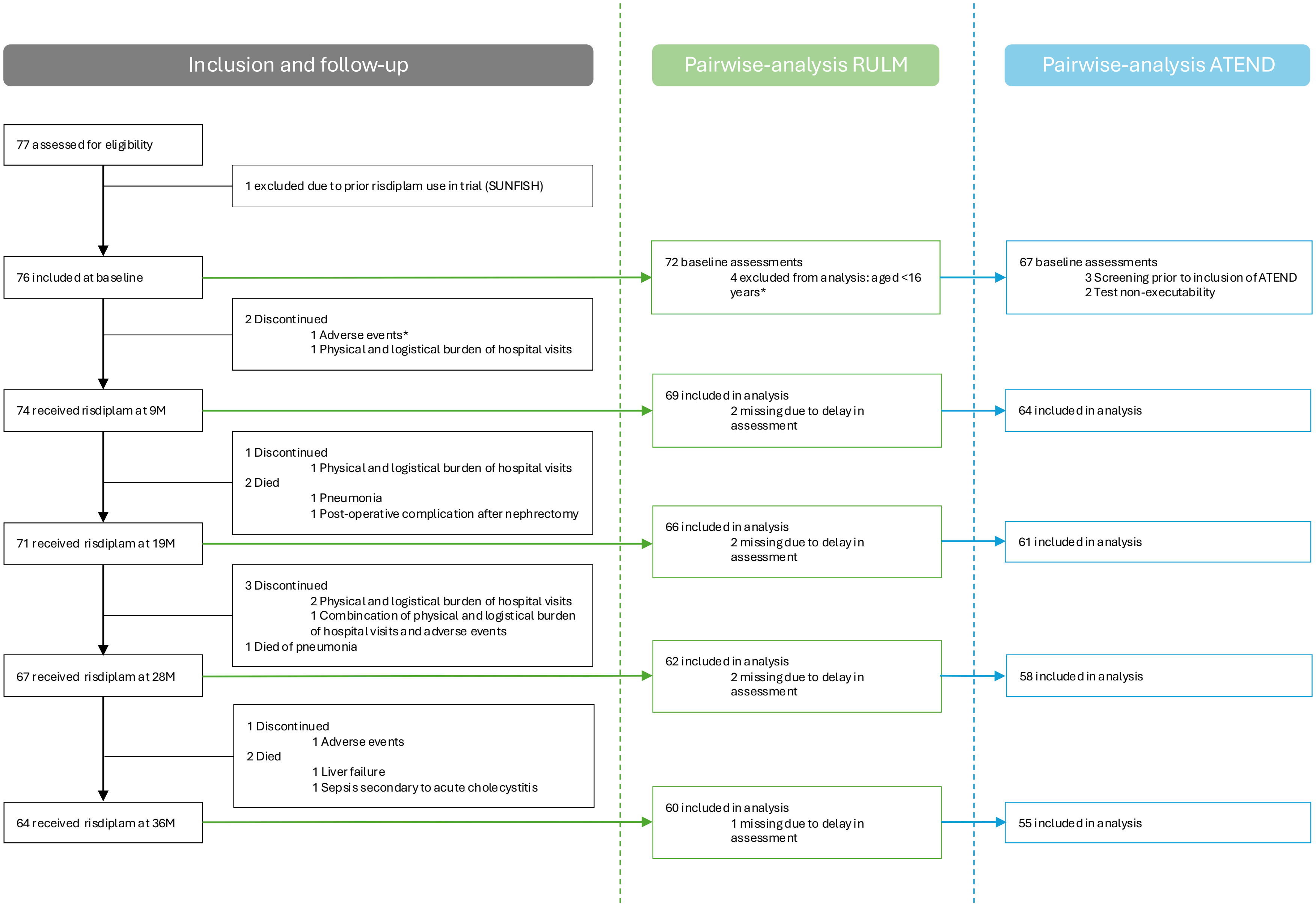

Supplementary figure S1. Study flow diagram detailing inclusion, exclusions, dropouts, and deaths, with inclusion in pairwise analysis of RULM (green) and ATEND (blue). *One patient who discontinued treatment before 9 months was excluded from the analysis; therefore, 6 discontinued patients are reported in the manuscript.

VII. Supplementary table S3: Changes in motor function scores

Supplementary table S3. Changes in motor function scores during risdiplam treatment compared to baseline

		**Screening**		**9M**				**19M**				**28M**				**36M**			
**Treatment duration – median months (IQR)**		0		9 (9–10)				19 (19–20)				28 (27–29)				36 (35–37)			
		**n**	**Median (IQR)**	**n**	**Median (IQR)**	**Median difference vs. baseline (IQR)**	**p value**	**n**	**Median (IQR)**	**Median difference vs. baseline (IQR)**	**p value**	**n**	**Median (IQR)**	**Median difference vs. baseline (IQR)**	**p value**	**n**	**Median (IQR)**	**Median difference vs. baseline (IQR)**	**p value**
**RULM score**	All patients	72	6 (2–15)	69	8 (2–16)	0 (0–1)	0·00064	66	8 (3–16)	0 (0–1)	0·029	62	5 (2–14)	0 (0–1)	ns	60	6 (2–14)	0 (–1–1)	ns
	SMA type 1c	8	2 (0–6)	8	2 (0–7)	0 (0–0)	ns	7	0 (0–6)	0 (–1–0)	ns	5	0 (0–5)	0 (–1–0)	ns	5	0 (0–2)	0 (–1–0)	ns
	SMA type 2a	43	4 (1–14)	40	6 (2–15)	0 (0–1)	0·0099	38	6 (2–15)	1 (0–1)	0·0079	38	5 (2–13)	0 (0–1)	ns	37	4 (2–12)	0 (–1–1)	ns
	SMA type 2b	21	14 (6–16)	21	14 (7–17)	0 (0–1)	ns	21	13 (5–17)	1 (0–2)	ns	19	12 (4–16)	0 (-2–1)	ns	18	13 (5–15)	0 (–1–1)	ns
**ATEND score**	All patients	67	24 (20–32)	69	26 (21–36)	1 (0–3)[1]	<0·0001	65	27 (20–35)	2 (0–4)[5]	<0·0001	62	26 (20–34)	2 (0–3)[8]	<0·0001	60	25 (20–33)	1 (0–3)[11]	0·0023
	SMA type 1c	5	21 (5–21)	8	14 (6–21)	0 (0–0)[2]	ns	7	8 (7–22)	–1 (–1–1)[6]	ns	5	8 (7–18)	–2 (–3–1)[9]	ns	5	7 (5–18)	–1 (–2–0)[12]	ns
	SMA type 2a	42	24 (20–30)	40	27 (23–33)	1 (0–3)[3]	0·00014	38	26 (21–34)	2 (0–3)[7]	0·00030	38	25 (20–32)	1 (0–3)[7]	0·0077	37	24 (20–32)	1 (–1–2)[13]	ns
	SMA type 2b	20	31 (22–34)	21	34 (24–37)	2 (1–3)[4]	0·00072	20	33 (26–37)	3 (1–4)	0·00029	19	33 (24–37)	3 (2–4)[10]	0·00068	18	32 (24–36)	2 (1–4)[14]	0·0027

1: n=64, 2: n=5, 3: n=39, 4: n=20; 5 n=61; 6: n=4; 7: n=37; 8: n=58, 9: n=3, 10: n=18, 11: n=55, 12: n=2, 13: n=36, 14: n=17.

Wilcoxon signed rank test was conducted pairwise, which resulted in the following number of included patients in the analysis of ATEND score: n=64 for baseline versus 9M (SMA type 1c: n=5, 2a: n=39, 2b: n=20); n=61 for baseline versus 19M (SMA type 1c: n=4, 2a: n=37, 2b: n=20); n=58 for baseline versus 28M (SMA type 1c: n=3, 2a: n=37, 2b: n=18), and n=55 for baseline versus 36M (SMA type 1c: n=2, 2a: n=36, 2b: n=17). We reported p values from the Wilcoxon signed rank test. Results were confirmed by paired t-test, except for RULM score analyses in all patients at 19M (paired t-test p = 0·057) and in the SMA type 2b subgroup at 9M (paired t-test p=0·036).

ATEND Adapted Test of Neuromuscular Disease; *IQR* Interquartile range; *ns* non-significant; *RULM* Revised Upper Limb Module; *SD* Standard deviation; *SMA* Spinal Muscular Atrophy

VIII. Supplementary figure S2: Changes in RULM score at 9M, 19M, 28M

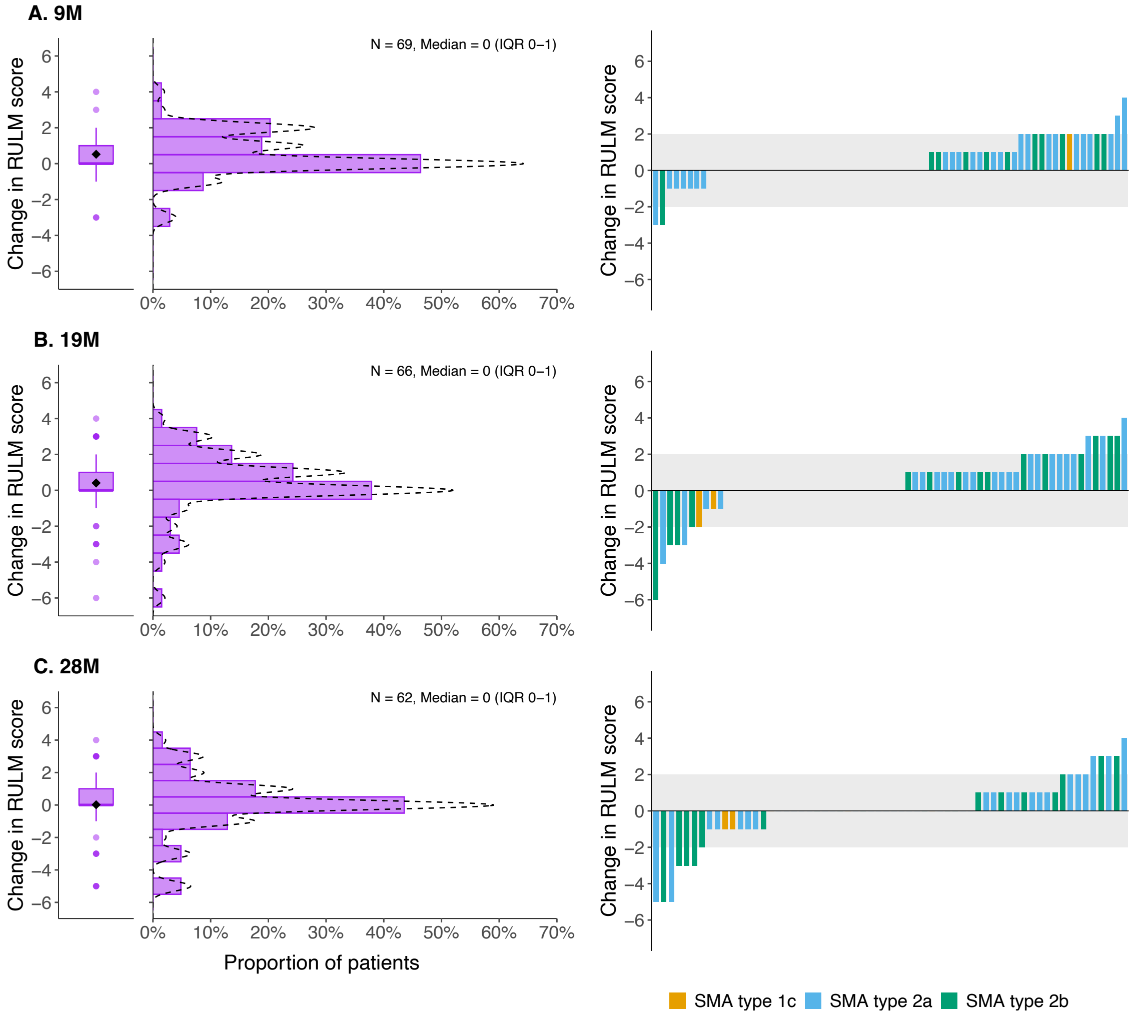

Supplementary figure S2. RULM score changes during treatment with risdiplam (9M, 19M and 28M). Histograms on the left show the proportion of patients (%) with specific change in scores (follow-up visit compared to baseline). The mean (diamond), median (central line), IQR (box), 1·5 x IQR (whiskers) and outliers (dots) are shown at the bottom of each histogram. The dashed line represents the kernel of distribution. Individual RULM score changes are depicted as bars. The grey area represents changes within ±2 points, which is considered day to day variation.[23] The empty space between improving patients and declining patients represents patients with change in score of 0 points. The median change in RULM score remained 0 for the whole follow-up period. We observed an improvement in RULM score of ≥2 points in 16, 15, and 9 (23, 23, and 15%) patients at 9M, 19M, and 28M, respectively. Improvement or stability in RULM score (≥0) was observed in 48, 43, and 35 (70, 65, and 57%) patients at 9M, 19M, and 28M, respectively. A decline in RULM score of ≥2 points was observed in 2, 7, and 7 (3, 11, and 11%) patients at 9M, 19M, and 28M, respectively. *IQR* Interquartile range; *RULM* Revised Upper Limb Module; *SMA* Spinal Muscular Atrophy

IX. Supplementary figure S3: Subgroup analysis (baseline RULM ≥5)

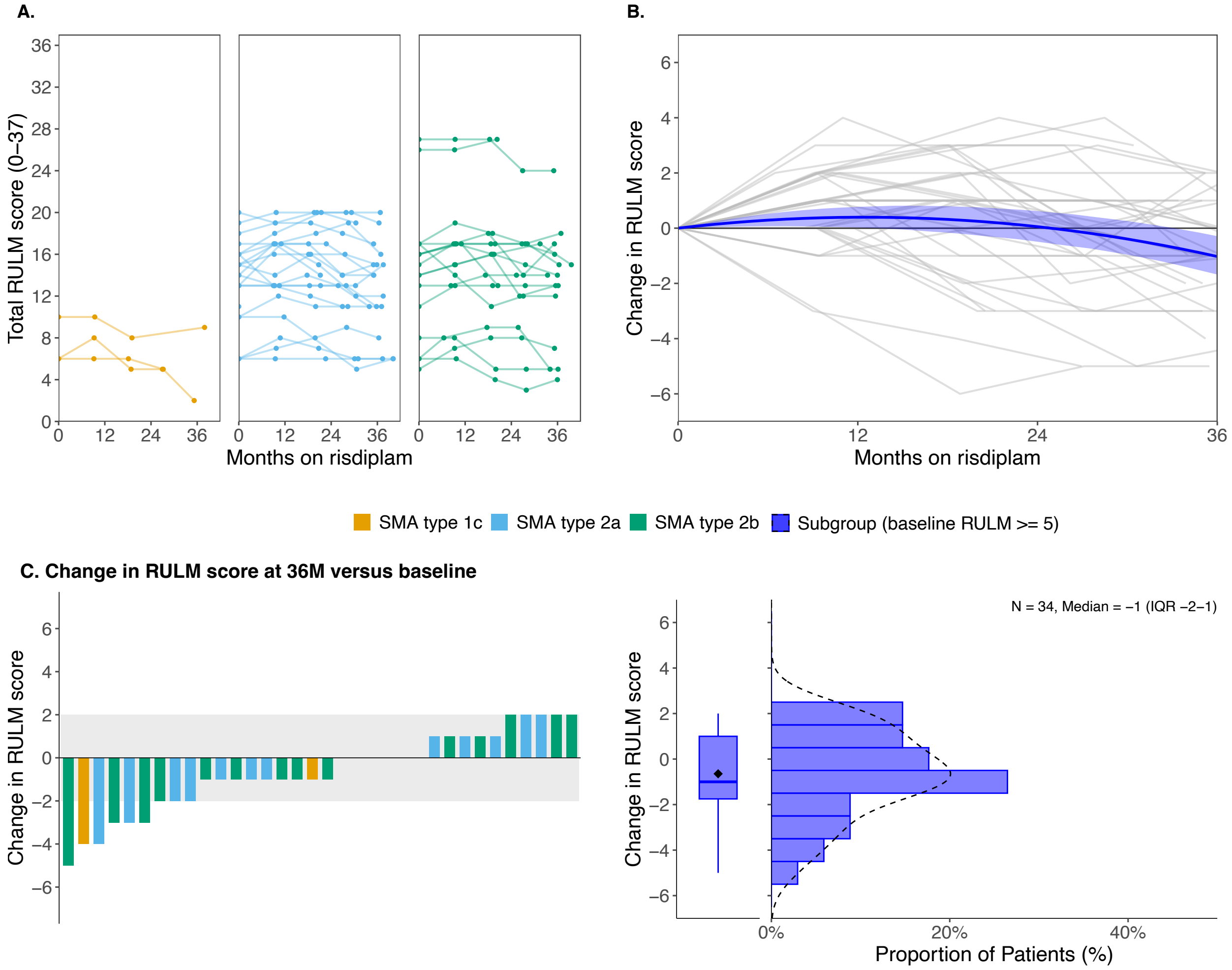

Supplementary figure S3. RULM scores during treatment with risdiplam in a subgroup of patients with baseline RULM score of ≥5. (AB) The spaghetti plot represents individual patient trajectories of the total RULM score over time stratified for SMA type (indicated by different colours) and delta RULM score (grey). **(A)** The dots represent the different timepoints for assessment during follow-up. **(B)** The dark blue line and dark blue shading reflect an estimation of the average change in RULM and the 95%CI, respectively. **(C)** Individual RULM score changes at 36M are depicted as bars; 6 patients without changes at 36M compared to baseline are reflected by the straight line. The grey area represents changes within ±2 points, which is considered day to day variation.[23] The histogram shows the proportion of patients (%) with specific score changes at 36M versus baseline. On the left, mean (diamond), median (line), IQR (box), 1·5×IQR (whiskers), and outliers (dots) are indicated. The dashed line shows the kernel density. At 36M, we observed an improvement in RULM score of ≥2 points in 5 (15%) patients, while the RULM score in 16 (47%) patients improved or remained stable. A decline in RULM score of ≥2 points was observed in 9 (26%) patients. *IQR* Interquartile range; *RULM* Revised Upper Limb Module; *SMA* Spinal Muscular Atrophy.

X. Supplementary figure S4: Association between PGIC and ATEND or RULM

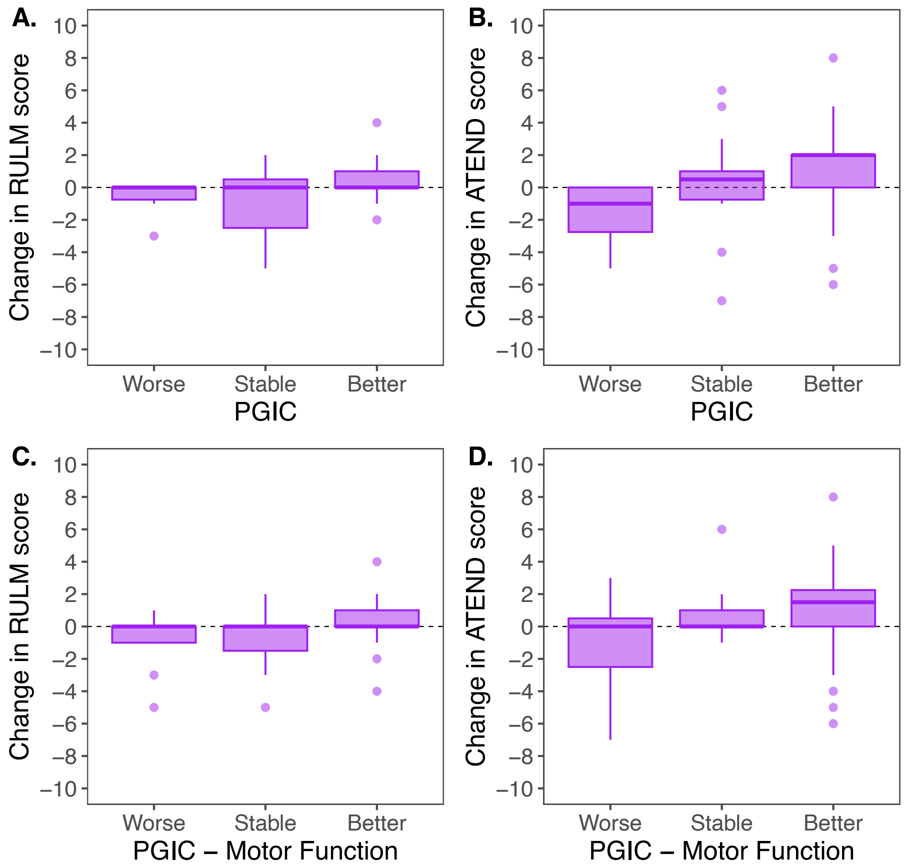

Supplementary figure S4. Association between patient reported changes and change in ATEND and RULM scores. The y-axis shows the change in RULM (panels A and C) and ATEND (panels B and D) motor function score compared to baseline and the x-axis shows the PGIC, categorized as worse (–3, –2, –1), stable (0), and better (+1, +2, +3). Kendall's rank correlation indicated a weak but statistically significant positive association between change in RULM score and motor PGIC (τ=0.270, p=0.020), and a somewhat stronger association for change in ATEND score (τ=0.286, p=0.014), which was similar for overall PGIC (τ=0.250, p= 0.032 and τ=0.318, p= 0.0066 for RULM and ATEND score, respectively). *ATEND* Adapted Test of Neuromuscular Diseases; *PGIC* Patient Global Impression of Change; *RULM* Revised Upper Limb Module; *SMA* Spinal Muscular Atrophy.

XI. Supplementary figure S5: Correlation ATEND and RULM over time

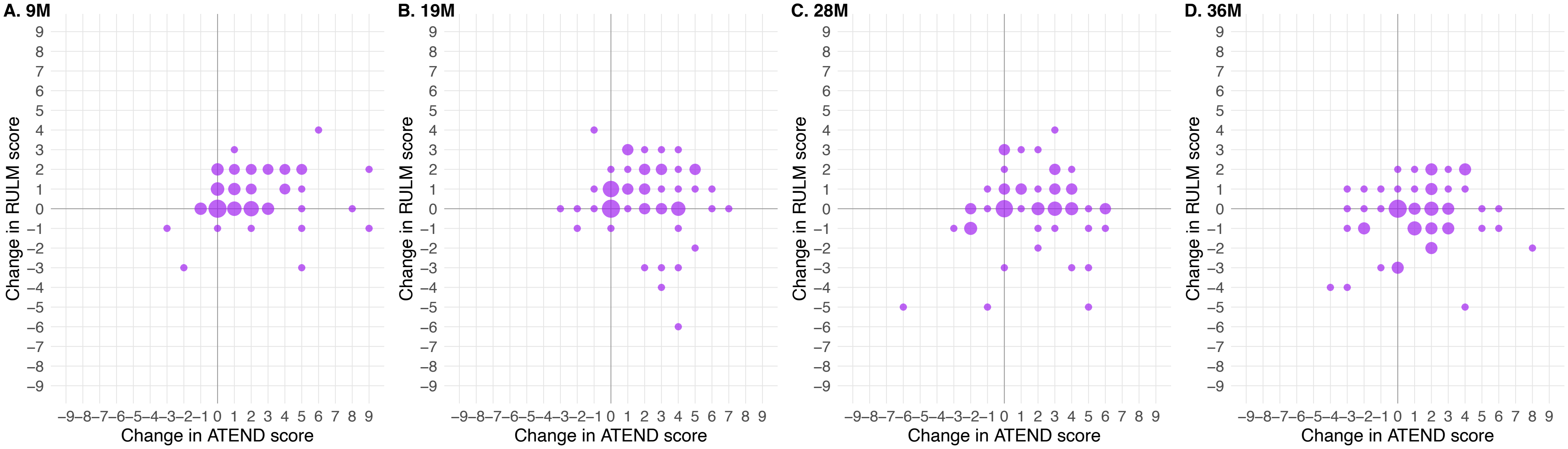

Supplementary figure S5. ATEND, but not RULM, captures improvement in motor function during risdiplam treatment. These dot plots visualise the correlation between change in RULM score as opposed to change in ATEND score after 9 months **(A)**, 19 months **(B)**, 28 months **(C)**, and 36 months **(D)** compared to baseline. The size of the dots depicts the patient count. In general, a change in RULM score is accompanied by a concomitant change in ATEND, demonstrating the existence of a correlation between the two. In addition, ATEND has an additive value in motor function assessment during treatment. This is visualised by the dots from -2 to 2 in RULM (y-axis), scoring outside this range on the ATEND (x-axis). Six patients with improving ATEND scores lost >2 RULM points during follow-up (9M: n=1, 19M: n=5, 28M: n=3, 36M: n=1). Conversely, only one patient gained >2 points on RULM while losing 1 point on ATEND at 19M. *ATEND* Adapted Test of Neuromuscular Diseases; *RULM* Revised Upper Limb Module

XII. Supplementary figure S6: Changes in ATEND score at 9M, 19M, 28M

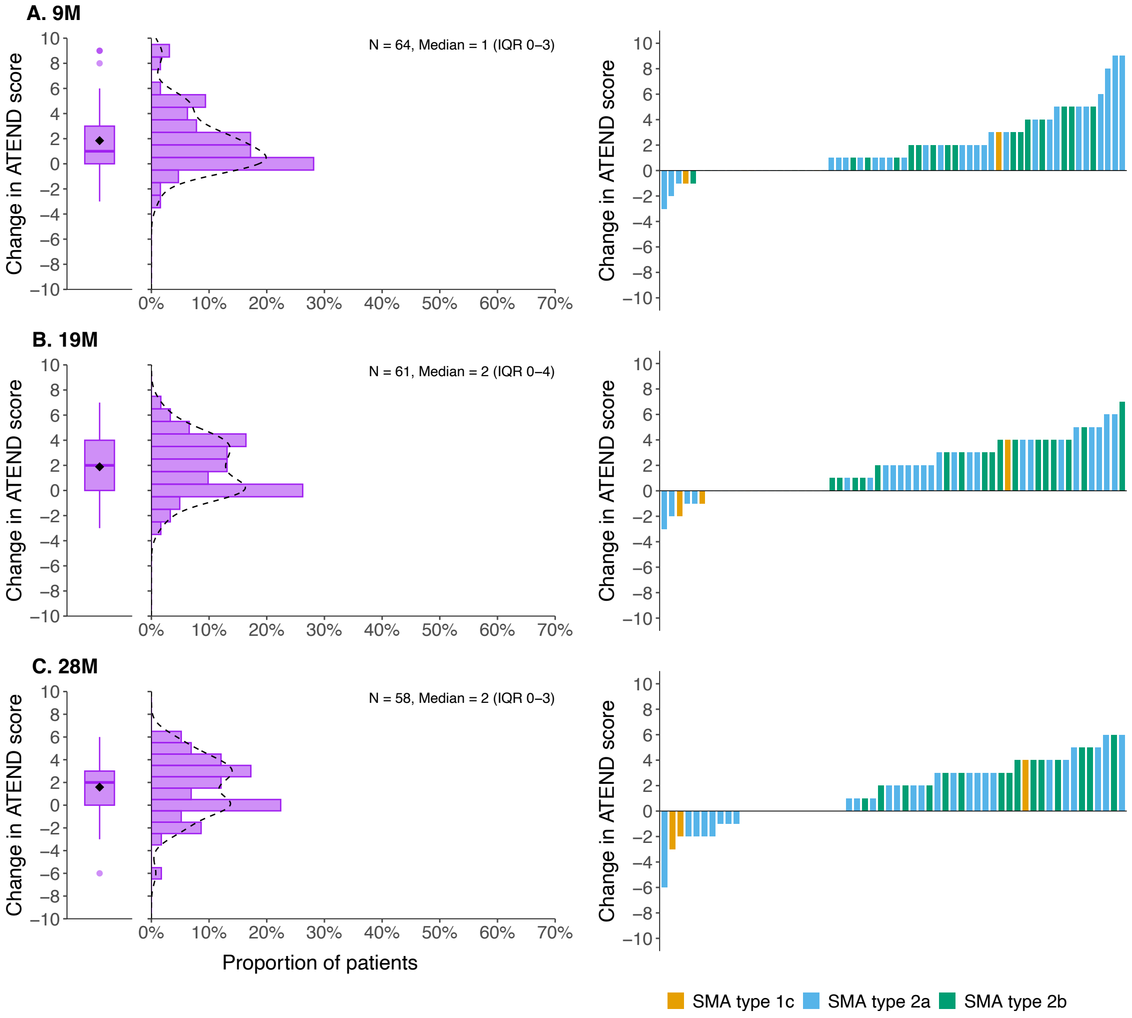

Supplementary figure S6. The greatest proportion of patients increased in ATEND score during risdiplam treatment (9M, 19M and 28M). Histograms on the left show the proportion of patients (%) with specific delta scores (follow-up visit compared to baseline). The mean (diamond), median (central line), IQR (box), 1·5 x IQR (whiskers) and outliers (dots) are shown at the bottom of each histogram. The dashed line represents the kernel of distribution. Individual ATEND score changes at 36M are depicted as bars. The empty space between improving patients and declining patients represents patients with delta score of 0. We observed increase in ATEND score of ≥1 points in 41, 39, and 35 (64, 64, and 60%) patients and either stable scores or increase (i.e., no points lost) in 59, 55, and 48 (92, 90, and 83%) patients at 9M, 19M, and 28M, respectively. A decline in ATEND score of ≥2 points was observed in 2, 3, and 7 (3, 5, and 12%) patients at 9M, 19M, and 28M, respectively. Median delta ATEND scores continued to be improved over time. *ATEND* Adapted Test of Neuromuscular Diseases; *IQR* Interquartile range; *SMA* Spinal Muscular Atrophy.

XIII. MCID estimation of the ATEND

Using the PGIC as external anchor, we estimated a MCID for ATEND at +1·0 points, based on the mean change in ATEND score in patients who reported at least ‘minimally improved’ overall well-being. Using the anchor-based distribution model, we estimated a MCID of +0·5 points, based on the optimal cut-off for discrimination between patients who reported ‘minimally improved’ or better overall well-being and patients who reported ‘no change’ or worse.

XIV. Supplementary table S4: hand grip strength

Supplementary table S4. Variability of hand grip strength during risdiplam treatment.

		Screening		9M			19M			28M			36M		
		**n**	**Median (IQR)**	**n**	**Median (IQR)**	**Median relative difference vs. baseline (IQR)**	**n**	**Median (IQR)**	**Median relative difference vs. baseline (IQR)**	**n**	**Median (IQR)**	**Median relative difference vs. baseline (IQR)**	**n**	**Median (IQR)**	**Median relative difference vs. baseline (IQR)**
**All patients**	Grip strength	55	1·00 (0·46–1·88)	53	1·17 (0·53–1·90)	4·3 (–7·9–25·6)	51	1·11 (0·52–1·94)	–5·7 (–19·7–10·4)[1]	41	1·09 (0·55–2·16)	1·5 (–16·3–16·0)[2]	45	1·09 (0·51–1·77)	2·4 (–12·2–13·7)[3]
	Pinch grip strength	71	0·432 (0·228–0·654)	66	0·508 (0·274–0·796)	8·1 (–10·6–27·6)	63	0·478 (0·252–0·948)	10·8 (–17·6–33·7)	33	0·483 (0·262–0·808)	–0·5(–17·1–14·6)	3	0·200 (0·130–0·354)	–39·5 (–46·0–43·6)
**SMA type 1c**	Grip strength	4	0·45 (0·34–0·54)	4	0·50 (0·40–0·58)	9·9 (7·4–22·8)	3	0·39 (0·21–0·53)	–11·4 (–34·3– –10·4)	3	0·45 (0·24–0·59)	–1·4 (–29·2–0·5)	2	0·31 (0·31–0·36)	90·6 (43·0–138·0)
	Pinch grip strength	7	0·224 (0·162–0·403)	7	0·180 (0·082–0·313)	–19·9 (–33·9– –12·4)	6	0·135 (0·075–0·266)	–7·4 (–37·5–22·2)	3	0·186 (0·134–0·381)	–19·5 (–26·7– –10·0)	2	0·284 (0·171–0·396)	37·2 (–7·6–82·0)
**SMA type 2a**	Grip strength	35	1·00 (0·49–1·83)	34	1·17 (0·56–1·71)	3·6 (–9·8–35·6)	33	1·02 (0·49–1·68)	–5·7 (–23·6–9·2)[4]	27	1·09 (0·55–1·85)	–0·2 (–17·2–6·7)[5]	30	1·07 (0·50–1·64)	5·4 (–13·6–14·3)[6]
	Pinch grip strength	43	0·354 (0·232–0·603)	39	0·468 (0·260–0·702)	7·7 (–11·7–27·3)	37	0·448 (0·238–0·946)	15·9 (–17·3–35·0)	18	0·395 (0·258–0·695)	–0·4 (–20·4–23·4)	1	0·200	–39·6
**SMA type 2b**	Grip strength	16	1·42 (0·93–2·58)	15	1·89 (0·96–2·57)	–4·2 (–6·9–22·0)	15	1·96 (0·91–2·63)	7·9 (–10·5–18·0)	11	2·06 (0·69–2·61)	17·2 (–7·4–32·0)	13	1·77 (0·96–2·63)	0·0 (–11·9–12·4)
	Pinch grip strength	21	0·607 (0·432–0·916)	20	0·773 (0·586–1·101)	15·1 (7·5–36·6)	20	0·682 (0·487–0·994)	8·0 (–9·0–30·6)	12	0·655 (0·467–1·096)	3·5 (–8·3–17·1)	0	··	··

1: n=50, 2: n=40, 3: n=44, 4: n=32, 5: n=26, 6: n=29

No statistical testing was performed due to extensive missing data (>20%) and high variability due to operational constraints, including test non-executability, inconsistent measurement settings, equipment unavailability, and unreliable test outcomes. Because missingness was linked to procedural factors rather than occurring at random, we reported only descriptive statistics (medians, IQRs) to avoid bias. Data is presented in absolute strength (kg) and relative difference (= (follow-up strength - baseline strength)/baseline strength). Data variability is highlighted by the broad IQR of relative difference over time.

Missing data grip strength: Measurements of 10 patients were excluded due to inconsistent MyoGrip settings. In addition, grip strength data was missing for 13% at screening (test non-executability (n=7)); for 11% at 9M (test non-executability (n=6)); for 14% at 19M (test non-executability (n=7)); for 32% at 28M (non-executable (n=7) and equipment unavailability (n=6)); and for 16% at 36M (test non-executability (n=5), equipment unavailability (n=1), and use of other equipment (n=1)).

Missing data pinch grip strength: Pinch grip strength data was missing for 1% at screening (test non-executability (n=1)); for 4% at 9M (test non-executability (n=3)); for 5% at 19M (test non-executability (n=3)); for 47% at 28M (test non-executability (n=5), equipment unavailability (n=23) and deemed unreliable by physiotherapist (n=1)); and for 95% at 36M (test non-executability (n=1), equipment unavailability (n=50), use of other equipment (n=1), and unknown (n=5)).

IQR Interquartile range; *SD* Standard deviation; *SMA* Spinal Muscular Atrophy

XV. Supplementary table S5: adverse events

Supplementary table S5. Adverse events during risdiplam by system organ class

	number of events (percentage of overall events)	number of patients (percentage of study population)	number of hospital admissions
**Overall adverse events**	**303**	**67 (93%)**	**37**
Infections and infestations	120 (40%)	49 (73%)	20
Gastrointestinal disorders	61 (20%)	32 (48%)	3
Skin and subcutaneous tissue disorders	20 (7%)	16 (24%)	0
Injury, poisoning and procedural complications	15 (5%)	12 (18%)	1
Surgical and medical procedures	12 (4%)	11 (16%)	4
Cardiac disorders	11 (4%)	7 (10%)	0
Renal and urinary disorders	11 (4%)	10 (15%)	3
Musculoskeletal and connective tissue disorders	7 (2%)	7 (10%)	1
General disorders and administration site conditions	6 (2%)	6 (9%)	0
Blood and lymphatic system disorders	6 (2%)	6 (9%)	0
Vascular disorders	6 (2%)	6 (9%)	0
Metabolism and nutrition disorders	6 (2%)	4 (6%)	0
Nervous system disorders	5 (2%)	4 (6%)	0
Hepatobiliary disorders	4 (1%)	4 (6%)	1
Oral and dental disorders	4 (1%)	4 (6%)	0
Endocrine disorders	3 (1%)	3 (4%)	2
Eye disorders	2 (1%)	2 (3%)	1
Neoplasms benign, malignant and unspecified	2 (1%)	2 (3%)	1
Immune system disorders	1 (0%)	1 (1%)	0
Respiratory, thoracic and mediastinal disorders	1 (0%)	1 (1%)	0

Adverse events were defined as events occurring or worsening after the first administration of risdiplam up to the last visit included in our study. Adverse events were coded using the Medical Dictionary for Regulatory Activities (MeDRA, version 6.0, ref: https://www.meddra.org/).

REFERENCES

1 Baranello G, Darras BT, Day JW, *et al.* Risdiplam in type 1 spinal muscular atrophy. *N Engl J Med* 2021; **384**: 915–23.

2 Masson R, Mazurkiewicz-Bełdzińska M, Rose K, *et al.* Safety and efficacy of risdiplam in patients with type 1 spinal muscular atrophy (FIREFISH part 2): secondary analyses from an open-label trial. *Lancet Neurol* 2022; **21**: 1110–9.

3 McCluskey G, Lamb S, Mason S, *et al.* Risdiplam for the treatment of adults with spinal muscular atrophy: Experience of the Northern Ireland neuromuscular service. *Muscle Nerve* 2023; **67**: 157–61.

4 Mercuri E, Baranello G, Boespflug-Tanguy O, *et al.* Risdiplam in types 2 and 3 spinal muscular atrophy: A randomised, placebo-controlled, dose-finding trial followed by 24 months of treatment. *Eur J Neurol* 2023; **30**: 1945–56.

5 Oskoui M, Day JW, Deconinck N, *et al.* Two-year efficacy and safety of risdiplam in patients with type 2 or non-ambulant type 3 spinal muscular atrophy (SMA). *J Neurol* 2023; **270**: 2531–46.

6 Sitas B, Hancevic M, Bilic K, Bilic H, Bilic E. Risdiplam real world data - looking beyond motor neurons and motor function measures. *J Neuromuscul Dis* 2024; **11**: 75–84.

7 Brakemeier S, Lipka J, Schlag M, Kleinschnitz C, Hagenacker T. Risdiplam improves subjective swallowing quality in non-ambulatory adult patients with 5q-spinal muscular atrophy despite advanced motor impairment. *J Neurol* 2024; **271**: 2649–57.

8 Iterbeke L, Claeys KG. Two-year Risdiplam treatment in adults with spinal muscular atrophy: improvements in motor and respiratory function, quality of life and fatigue. *Neuromuscul Disord* 2025; **52**: 105397.

9 Keritam O, Erdler M, Fasching B, *et al.* Efficacy and safety of risdiplam in adults with 5q-associated spinal muscular atrophy: a nationwide observational cohort study in Austria. *EClinicalMedicine* 2025; **88**: 103536.

10 Parmova O, Prasil K, Mokra L, *et al.* A real-world, multicentre, epidemiological study in Czech and Slovak adults with spinal muscular atrophy treated with risdiplam. *Sci Rep* 2026; **16**. DOI:10.1038/s41598-026-37462-6.

11 Darras BT, Masson R, Mazurkiewicz-Bełdzińska M, *et al.* Risdiplam-treated infants with type 1 spinal muscular atrophy versus historical controls. *N Engl J Med* 2021; **385**: 427–35.

12 Mercuri E, Deconinck N, Mazzone ES, *et al.* Safety and efficacy of once-daily risdiplam in type 2 and non-ambulant type 3 spinal muscular atrophy (SUNFISH part 2): a phase 3, double-blind, randomised, placebo-controlled trial. Lancet Neurol. 2022; **21**: 42–52.

13 Duong T, Tang W, Nelson L, *et al.* P.47 Adaptive test for neuromuscular disorders: Design of a wheelchair-based assessment. *Neuromuscul Disord* 2022; **32**: S61.

14 Nelson L, Tang W, Pasternak A, Glanzman A, Muni Lofra R, Duong T. 218P Inter-rater reliability of adapted test of neuromuscular disorders (ATEND) wheelchair-based assessment. *Neuromuscul Disord* 2024; **43**: 104441.60.

15 Duong T, Muni-Lofra R, Pasternak A, *et al.* 194P Longitudinal assessment of the Adapted Test for Neuromuscular Disease (ATEND) in individuals living with spinal muscular atrophy (SMA). *Neuromuscul Disord* 2024; **43**: 104441.445.

16 Merlini L, Mazzone ES, Solari A, Morandi L. Reliability of hand-held dynamometry in spinal muscular atrophy. *Muscle Nerve* 2002; **26**: 64–70.

17 Febrer A, Rodriguez N, Alias L, Tizzano E. Measurement of muscle strength with a handheld dynamometer in patients with chronic spinal muscular atrophy. *J Rehabil Med* 2010; **42**: 228–31.

18 Werlauff U, Fynbo Steffensen B. The applicability of four clinical methods to evaluate arm and hand function in all stages of spinal muscular atrophy type II. *Disabil Rehabil* 2014; **36**: 2120–6.

19 Guy W. Clinical Global Impressions. In: ECDEU assessment manual for psychopharmacology - Revised 76-338. Rockville, Maryland: U. S. Dept. of Health, Education, and Welfare, Public Health Service, Alcohol, Drug Abuse, and Mental Health Administration, National Institute of Mental Health, Psychopharmacology Research Branch, Division of Extramural Research Programs, 1976: 218–22.

20 Rampakakis E, Ste-Marie PA, Sampalis JS, Karellis A, Shir Y, Fitzcharles M-A. Real-life assessment of the validity of patient global impression of change in fibromyalgia. *RMD Open* 2015; **1**: e000146.

21 Scott W, McCracken LM. Patients' impression of change following treatment for chronic pain: global, specific, a single dimension, or many? *J Pain* 2015; **16**: 518–26.

22 Vázquez-Costa JF, Povedano M, Nascimiento-Osorio AE, *et al.* Nusinersen in adult patients with 5q spinal muscular atrophy: A multicenter observational cohorts' study. *Eur J Neurol* 2022; **29**: 3337–46.

23 Coratti G, Pera MC, Montes J, *et al.* Different trajectories in upper limb and gross motor function in spinal muscular atrophy. *Muscle Nerve* 2021; **64**: 552–9.
